# In reply to the letter to the editor regarding “The Relationship Between Serum Vitamin D and Fracture Risk in the Elderly: A Meta-Analysis”

**DOI:** 10.1186/s13018-020-01820-5

**Published:** 2020-08-14

**Authors:** Ning Wang, Jindou Ji, Shengwen Yu, Jinlei Chang, Yungang Chen, Bo Yu

**Affiliations:** 1 Shandong University of Traditional Chinese Medicine, Jinan, 250014 China; 2 Department of Orthopaedics, Affiliated Hospital of Shandong University of Traditional Chinese Medicine, Jinan, 250014 China

Dear Editor,

We wish to thank all of the reviewers for their thought-provoking and insightful comments about our recent meta-analysis published in the journal [1]. Our answers to the questions raised are as follows:

First, at present, there is no conclusion about the database to be searched. The two databases we searched (PubMed and Embase) can find most of the literature. Many meta-analysis also searched only two or three databases [2–12].

Second, registration is not a necessary condition for meta-analysis. On the contrary, most meta-analyses are not registered in PROSPERO. Compared with the significance of the research itself, whether it is registered in the Cochrane Library or PROSPERO is not a matter of special concern. We strictly followed the steps of PRISMA during the research process. According to the relevant requirements of JOSR, the author is required to provide registration information about the system review, including the registration number (if any), and we have clearly indicated our registration status in our Abstract.

Third, participants from Swanon et al. and Cauley et al. are indeed from the same research cohort, but the number of people included in each study is different. More importantly, adjustment factors for the final results are quite different, and the final results remain unchanged even when we exclude one of the studies.

Fourth, compared with previous meta-analyses conducted on this topic, our research sets standards based on bone turnover rate and stricter age limits, which was why we conducted a subgroup analysis of gender. We did feel it was necessary to conduct analysis on BMI as a previous meta-analysis already studied BMI and showed that low BMI under any conditions is one of the risk factors for elderly fractures [13, 14].

Finally, thanks again for the comments you made on this article, and we hope that our reply helps answer your questions.

## Data Availability

The datasets used and/or analyzed during the current study are available from the corresponding author on reasonable request.
